# The crystal structures of two chalcones: (2*E*)-1-(5-chloro­thio­phen-2-yl)-3-(2-methyl­phen­yl)prop-2-en-1-one and (2*E*)-1-(anthracen-9-yl)-3-[4-(propan-2-yl)phen­yl]prop-2-en-1-one

**DOI:** 10.1107/S2056989016011592

**Published:** 2016-07-19

**Authors:** Marisiddaiah Girisha, Hemmige S. Yathirajan, Jerry P. Jasinski, Christopher Glidewell

**Affiliations:** a Department of Studies in Chemistry, University of Mysore, Manasagangotri, Mysuru 570 006, India; b Department of Chemistry, Keene State College, 229 Main Street, Keene, NH 03435-2001, USA; c School of Chemistry, University of St Andrews, Fife KY16 9ST, UK

**Keywords:** crystal structure, synthesis, chalcones, mol­ecular structure, mol­ecular conformation, conformational disorder, hydrogen bonding, supra­molecular assembly

## Abstract

In the crystal of compound (I), C_14_H_11_ClOS, mol­ecules are linked by C—H⋯O hydrogen bonds to form simple *C*(5) chains. Compound (II), C_26_H_22_O, crystallizes with *Z*′ = 2 in space group *P*-1; one of the mol­ecules is fully ordered but the other is disordered over two sets of atomic sites having occupancies 0.644 (3) and 0.356 (3). The two disordered components differ from one another in the orientation of the isopropyl substituents, and both differ from the ordered mol­ecules in the arrangement of the central propenone spacer unit, so that the crystal of (II) contains three distinct conformers. The ordered and disordered conformers each form a *C*(8) chain built from a single type of C—H⋯O hydrogen bond but those formed by the disordered conformers differ from that formed by the ordered form.

## Abstract 2

Mol­ecules of (2*E*)-1-(5-chloro­thio­phen-2-yl)-3-(2-methyl­phen­yl)-prop-2-en-1-one (I) are linked into simple hydrogen-bonded *C*(5) chains, while three distinct conformers of (2*E*)-1-(anthracen-9-yl)-3-[4-(propan-2-yl)phen­yl]prop-2-en-1-one (II) co-exist in the crystal but each conformer forms hydrogen-bonded *C*(8) chains, albeit of two different types.

## Chemical context   

Chalcones, *R* ^1^—C(=O)—CH=CH—*R* ^2^, are versatile inter­mediates in synthesis (Baktır *et al.*, 2011 ▸; Samshuddin *et al.*, 2011 ▸, 2012 ▸, 2014 ▸; Nayak *et al.*, 2014 ▸; Salian *et al.*, 2015 ▸; Mohan *et al.*, 2016 ▸). Compounds of this class also exhibit a wide range of biological activity, including anti-bacterial (Tran *et al.*, 2012 ▸), anti-cancer (Syam *et al.*, 2012 ▸; Kumar *et al.*, 2014 ▸), anti-fungal (López *et al.*, 2001 ▸), anti-inflammatory (Fang *et al.*, 2015 ▸), anti-malarial (Agarwal *et al.*, 2005 ▸) and anti­tubercular activities (Dimmock *et al.*, 1999 ▸). Accordingly, the synthesis and characterization of new examples of this type is of inter­est and potentially of value and herein we report on the synthesis and crystal structures of two further examples; (2*E*)-1-(5-chloro­thio­phen-2-yl)-3-(2-methyl­phen­yl)-prop-2-en-1-one (I), and (2*E*)-1-(anthracen-9-yl)-3-[4-(propan-2-yl)phen­yl]prop-2-en-1-one (II). Compounds (I) and (II) were prepared by base-induced condensation of an aryl aldehyde, 2-methyl­benzaldehyde in the case of (I) or 4-iso­propyl­benzaldehyde for (II) with, respectively, 2-acetyl-5-chloro­thio­phene or 9-acetyl­anthracene.

## Structural commentary   

**Figure 1:**
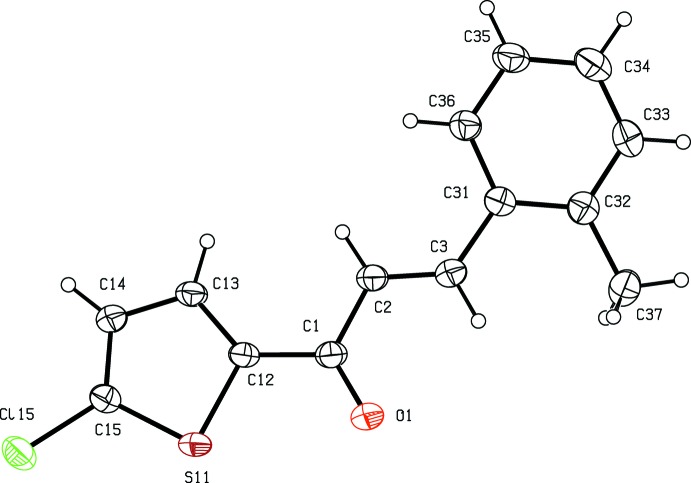
The mol­ecular structure of compound (I), with atom labelling and displacement ellipsoids drawn at the 30% probability level.

In the mol­ecule of compound (I), Fig. 1 ▸, the central spacer unit comprising the atoms (C12,C1,C2,C3,C31) is effectively planar: the maximum deviation from the mean plane of these atoms is 0.21 (2) Å, with an r.m.s. deviation of 0.025 Å. The heterocyclic ring is nearly co-planar with the spacer unit, making with it a dihedral angle of 1.41 (1)°. The dihedral angles between the phenyl group and the spacer unit, and between the two rings are 10.95 (11) and 9.81 (10)°, respectively. The bond distances within the mol­ecule of (I) show clearly the localized double bond between atoms C2 and C3, and the distances within the thio­phene ring clearly rule out the possibility of any orientational disorder of the type sometimes found in thio­phene rings (Cobo *et al.*, 2005 ▸; Trilleras *et al.*, 2005 ▸, 2009 ▸; Insuasty *et al.*, 2014 ▸).
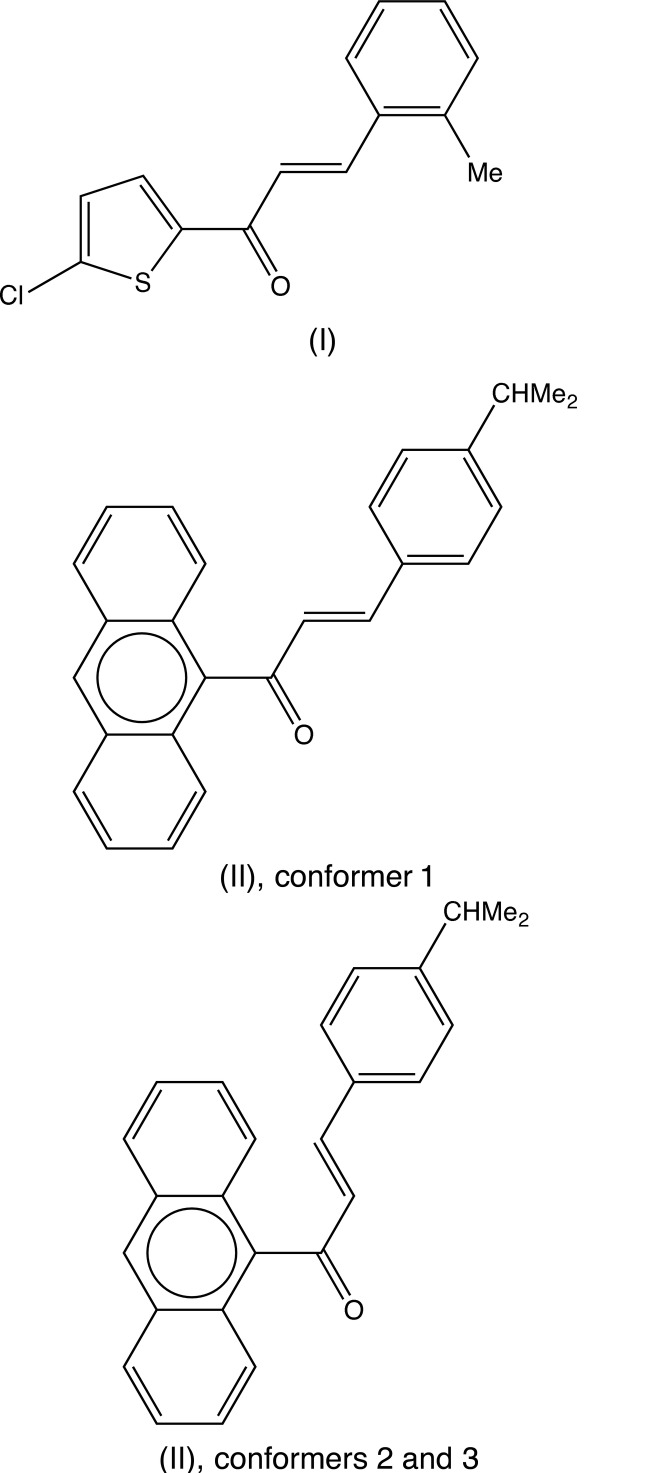


**Figure 2:**
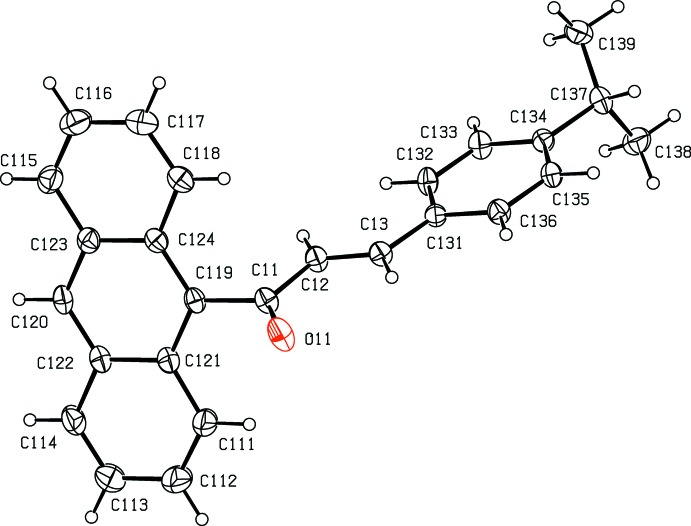
The mol­ecular structure of conformer 1 in compound (II), with atom labelling and displacement ellipsoids drawn at the 30% probability level.

**Figure 3:**
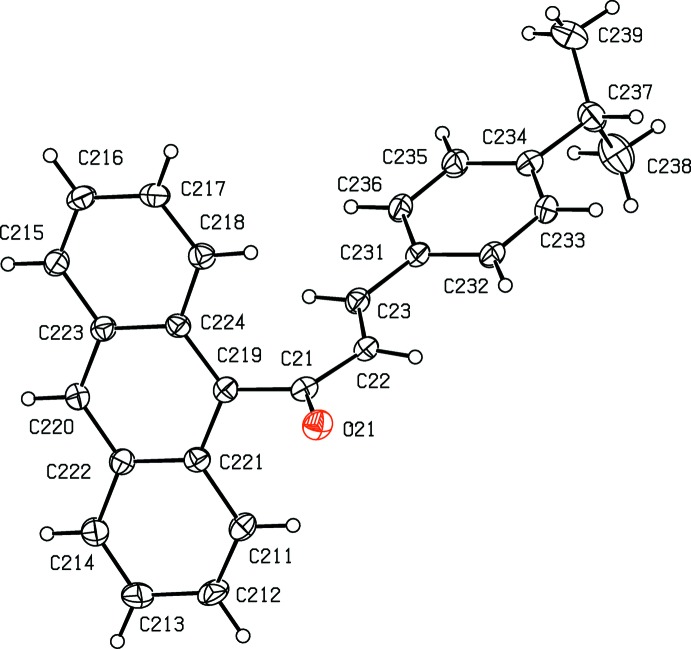
The mol­ecular structure of the major-disorder component, conformer 2 having occupancy 0.644 (3), in compound (II), with atom labelling and displacement ellipsoids drawn at the 30% probability level.

**Figure 4:**
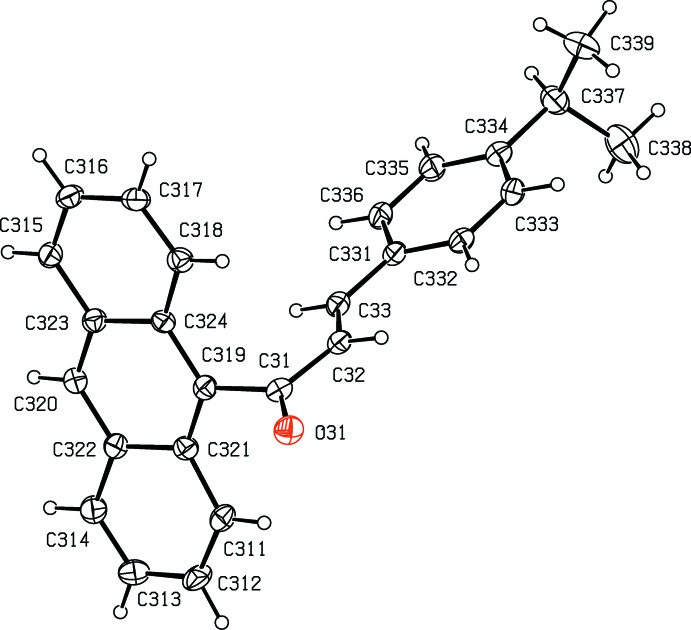
The mol­ecular structure of the minor-disorder component, conformer 3 having occupancy 0.356 (3), in compound (II), with atom labelling and displacement ellipsoids drawn at the 30% probability level.

Compound (II) crystallizes with *Z*′ = 2 in space group *P* 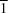. The mol­ecule containing atom O11 (Fig. 2 ▸) is fully ordered, but the other mol­ecule is disordered over two sets of atomic sites: the major-disorder component containing atom O21 (Fig. 3 ▸) has occupancy 0.644 (3) while the minor-disorder component containing atom O31 (Fig. 4 ▸) has occupancy 0.356 (3). All three forms exhibit different conformations, as discussed below, and it will be convenient to refer to the mol­ecules containing atoms O11, O21 or O31 as conformers of types 1, 2 or 3, respectively.

In the fully ordered mol­ecule containing atom O11 the torsional angle C119—C11—C12—C13 is 177.72 (16)° whereas in the two disordered components containing atoms O21 and O31 the values of the corresponding torsional angles C*n*19—C*n*1—C*n*2—C*n*3 are 11 (3)° and 12 (5)° for *n* = 2 and 3 respectively, corresponding to a rotation of approximately 180° about the bond C*n*1—C*n*2 in conformers 2 and 3 as compared with conformer 1. In addition, in conformers 2 and 3 the torsional angles C*n*33—C*n*34—C*n*37—H*n*37 are −14° and −170° for *n* = 2 and 3, respectively, so that the orientation of the CHMe_2_ group in these two forms differs by a rotation of approximately 180° about the bond C*n*34—C*n*37; the corresponding value in conformer 1 is *ca* 162°. Hence three different conformations of compound (II) co-exist in the same crystal (*cf.* Figs. 2 ▸–4) with relative abundances 1.000:0.644 (3):0.356 (3) in the crystal selected for data collection. Conformer 1 thus differs from conformers 2 and 3 in the arrangement of the central spacer unit, while conformers 1 and 3 exhibit similar orientations of the isopropyl unit relative to the adjacent phenyl ring, but different from that in conformer 2.

In each conformer, the central spacer unit encompassing atoms C*n*19,C*n*1,C*n*2,C*n*3,C*n*31 is effectively planar with r.m.s. deviations from the mean planes of 0.011, 0.036 and 0.043 Å for *n* = 1–3, respectively. This spacer unit makes dihedral angles with the central ring of the anthracene unit of 67.70 (11), 65.7 (5) and 71.7 (10)° for *n* = 1–3, respectively, and the corresponding dihedral angles with the adjacent aryl rings C*n*31—C*n*36 are 6.26 (18), 1.5 (11) and 7(2)°, respectively. These values confirm that the principal difference between conformer 1 and conformers 2 and 3 is simply a rotation about the bond C*n*1—C*n*2.

Within each of the anthracene units, the distances C*n*11—C*n*12, C*n*13—C*n*14, C*n*15—C*n*16 and C*n*17—C*n*18 are very much shorter than the other C—C bonds in these units, while the C—C distances in the central rings show rather little variation. These observations are consistent with an electronic structure for the anthracene units where a central ring displaying aromatic delocalization is flanked by two isolated diene units (Glidewell & Lloyd, 1984 ▸,1986 ▸).

## Supra­molecular features   

**Figure 5:**
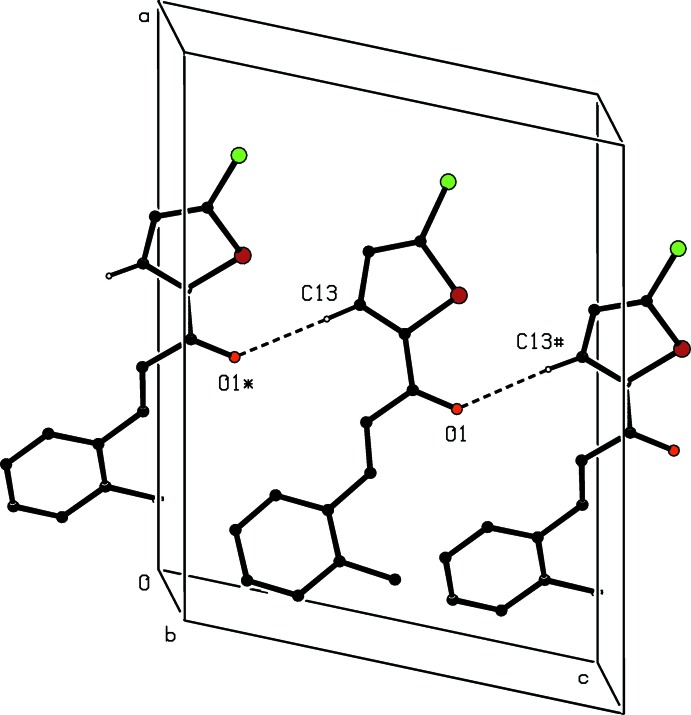
Part of the crystal structure of compound (I), showing the formation of a hydrogen-bonded *C*(5) chain parallel to [001]. Hydrogen bonds are shown as dashed lines and, for the sake of clarity, the H atoms not involved in the motif shown are omitted. The atoms marked with an asterisk (*) or a hash (#) are at the symmetry positions (*x*, 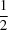 − *y*, −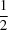 + *z*) and (*x*, 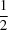 − *y*, 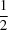 + *z*), respectively.

**Figure 6:**
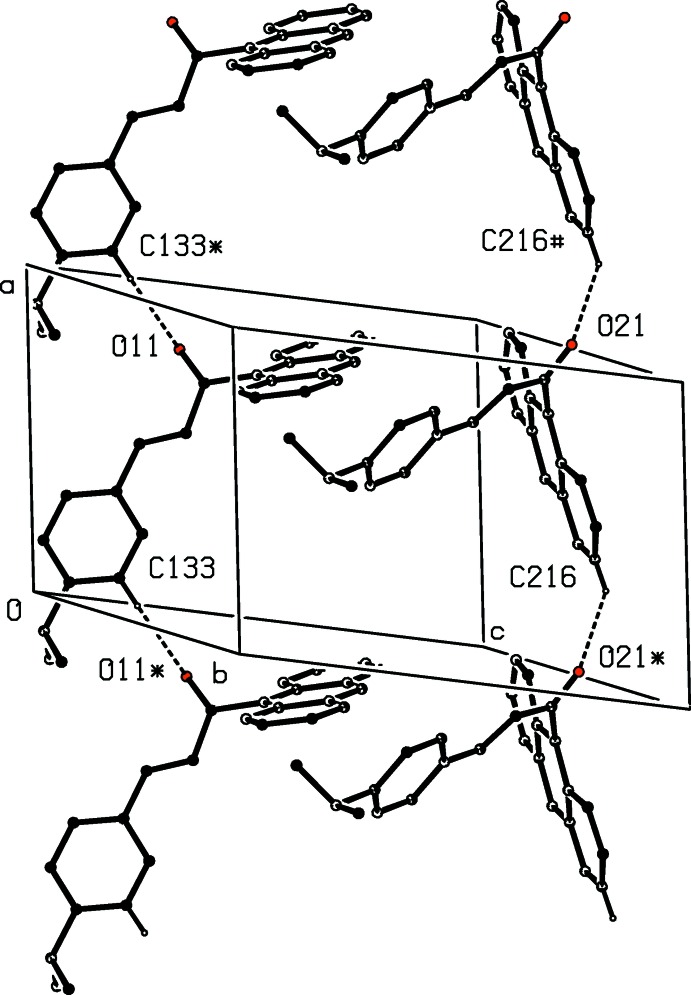
Part of the crystal structure of compound (II), showing the formation of two different types of hydrogen-bonded *C*(8) chain. Hydrogen bonds are shown as dashed lines and, for the sake of clarity, the minor-disorder component and the H atoms not involved in the motifs shown have been omitted. The atoms marked with an asterisk (*) or a hash (#) are at the symmetry positions (−1 + *x*, *y*, *z*) and (1 + *x*, *y*, *z*), respectively.

In the crystal of compound (I), mol­ecule related by a *c*-glide plane are linked by a single C—H⋯O hydrogen bond (Table 1 ▸) to form a *C*(5) chain running parallel to the [001] direction (Fig. 5 ▸). In the crystal of compound (II), mol­ecules are also linked into chains by C—H⋯O hydrogen bonds (Table 2 ▸), but the chains formed by the ordered and disordered forms are different, in that in the chain of ordered mol­ecules the donor is a phenyl C—H unit, while in the disordered forms the donors are part of the anthracene units. In both types of chain mol­ecules related by translation form *C*(8) chains running parallel to the [100] direction (Fig. 6 ▸). In addition, inversion-related pairs of the chains built from the disordered components are weakly linked by C—H⋯π inter­actions (Table 2 ▸).

## Database survey   

The structures of a number of chalcones containing substituted thio­phene units, and thus closely related to compound (I) have been reported recently (Naik, Shettigar *et al.*, 2015 ▸; Naik, Yathirajan *et al.*, 2015 ▸). There are no hydrogen bonds of any kind in the crystals of the isostructural compounds (2*E*)-1-(5-chloro­thio­phen-2-yl)-3-(4-ethyl­phen­yl)prop-2-en-1-one and (2*E*)-1-(5-bromo­thio­phen-2-yl)-3-(4-ethyl­phen­yl)prop-2-en-1-one, but in the isostructural compounds (2*E*)-1-(5-chloro­thio­phen-2-yl)-3-(4-eth­oxy­phen­yl)prop-2-en-1-one and (2*E*)-1-(5-bromo­thio­phen-2-yl)-3-(4-eth­oxy­phen­yl)prop-2-en-1-one the mol­ecules are linked by C—H⋯O hydrogen bonds to form simple *C*(7) chains, while *C*(5) chains are present in the structure of (2*E*)-1-(5-bromo­thio­phen-2-yl)-3-(3-meth­oxy­phen­yl)prop-2-en-1-one. In the structure of (2*E*, 2*’E*)-3, 3′-(1,3-phenyl­ene)-bis­(1-(anthracene-9-yl)prop-2-en-1-one), which is related to compound (II), inversion-related pairs of mol­ecules are linked by multiple C—H⋯O hydrogen bonds to form centrosymmetric dimers (Kant *et al.*, 2015 ▸). In the recently reported structure of (2*E*)-3-(2,4-di­chloro­phen­yl)-1-(2-meth­oxy­phen­yl)prop-2-en-1-one (Salian *et al.*, 2016 ▸), there are no hydrogen bonds of any kind, but the mol­ecules are linked into chains by π–π stacking inter­actions.

## Synthesis and crystallization   

For the synthesis of compound (I), a solution of 2-methyl­benzaldehyde (0.075 g, 0.625 mol) in methanol (20 ml) was added to solution of with 2-acetyl-5-chloro­thio­phene (0.100 g, 0.625 mol) in methanol (10 ml) and to this mixture was added aqueous sodium hydroxide solution (40% *w*/*v*, 5 ml). The reaction mixture was then stirred at 301 K for 4 h, when the resulting solid product was collected by filtration, washed with cold water and dried. Crystals suitable for single-crystal X-ray diffraction were grown by slow evaporation, at ambient temperature, of a solution in acetone-di­methyl­formamide (1:1, *v*/*v*): m. p. 387–389 K. For the synthesis of compound (II), aqueous sodium hydroxide solution (10%, *w*/*v*, 15 ml) was added to a mixture of 4-iso­propyl­benzaldehyde (1.5 ml, 0.01 mol) and 9-acetyl­anthracene (2.2 g, 0.01 mol) in ethanol (50 ml), and the resulting mixture was stirred at 278 K for 3 h. The resulting solid product was collected by filtration and recrystallized from ethanol solution: m.p. 369–371 K. Crystals suitable for single-crystal X-ray diffraction were grown by slow evaporation, at ambient temperature, of a solution in di­methyl­formamide.

## Refinement   

Crystal data, data collection and structure refinement details are summarized in Table 3 ▸. It was obvious from an early stage in the refinement of compound (II) that one of the two independent mol­ecules was disordered over two sets of atomic sites having unequal occupancies. For the minor-disorder component the bonded distances and the one-angle non-bonded distances were restrained to be the same as the corresponding distances in the major component, subject to s.u.s of 0.005 and 0.01 Å respectively. In addition, the anisotropic displacement parameters for corresponding atomic pairs of atomic sites in the two disorder components were constrained to be identical and, subject to these conditions the occupancies for the two components refined to values of 0.645 (4) and 0.355 (4). The H atoms in all but the minor-disorder component of compound (II) were located in difference maps and then treated as riding atoms in geometrically idealized positions with C—H distances 0.93 Å (alkenyl, aromatic and heteroaromatic), 0.96 Å (meth­yl) or 0.98 Å (aliphatic C—H) and with *U*_iso_(H) = *kU*_eq_(C) where *k* = 1.5 for the methyl groups, which were permitted to rotate but not to tilt, and 1.2 for all other H atoms. The H atoms in the minor-disorder component were included in the refinement in calculated positions under exactly the same conditions. For compound (II), 16 bad outliers of low intensity were omitted from the final refinements. In the final analysis of variance for compound (I) there was a fairly large value, 2.583, of *K* = mean (*F*_o_^2^)/mean (*F*_c_^2^) for the group of 287 very weak reflections having *F*_c_/*F*_c_(max) in the range 0.000 < *F*_c_/*F*_c_(max) < 0.006. For compound (II), there was a large value of *K*, 10.808, for the group of 733 very weak reflections having *F*_c_/*F*_c_(max) in the range 0.000 < *F*_c_/*F*_c_(max) < 0.005.

## Supplementary Material

Crystal structure: contains datablock(s) global, I, II. DOI: 10.1107/S2056989016011592/su5313sup1.cif

Structure factors: contains datablock(s) I. DOI: 10.1107/S2056989016011592/su5313Isup2.hkl

Structure factors: contains datablock(s) II. DOI: 10.1107/S2056989016011592/su5313IIsup3.hkl

Supporting information file. DOI: 10.1107/S2056989016011592/su5313Isup4.cml

Supporting information file. DOI: 10.1107/S2056989016011592/su5313IIsup5.cml

CCDC references: 1494027, 1494026

Additional supporting information:  crystallographic information; 3D view; checkCIF report

## Figures and Tables

**Table 1:** Hydrogen-bond geometry (Å, °) for (I)

*D*—H⋯*A*	*D*—H	H⋯*A*	*D*⋯*A*	*D*—H⋯*A*
C13—H13⋯O1^i^	0.93	2.55	3.467 (2)	169

Symmetry code: (i) 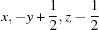.

**Table 2:** Hydrogen-bond geometry (Å, °) for (II) *Cg*1 and *Cg*2 are the centroids of rings (C111–C114/C122/C111) and (C114–C118/C124/C113), respectively.

*D*—H⋯*A*	*D*—H	H⋯*A*	*D*⋯*A*	*D*—H⋯*A*
C133—H133⋯O11^i^	0.93	2.49	3.336 (2)	151
C216—H216⋯O21^i^	0.93	2.61	3.41 (2)	144
C216—H216⋯O31^i^	0.93	2.58	3.36 (4)	142
C316—H316⋯O21^i^	0.93	2.51	3.30 (3)	144
C316—H316⋯O31^i^	0.93	2.47	3.25 (4)	142
C233—H233⋯*Cg*1^ii^	0.93	2.64	3.355 (4)	134
C236—H236⋯*Cg*2^iii^	0.93	2.75	3.519 (4)	140
C336—H336⋯*Cg*2^iii^	0.93	2.80	3.354 (7)	119

Symmetry codes: (i) 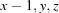; (ii) 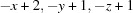; (iii) 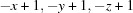.

**Table 3:** Experimental details

	(I)	(II)
Crystal data		
Chemical formula	C_14_H_11_ClOS	C_26_H_22_O
*M*_r_	262.74	350.44
Crystal system, space group	Monoclinic, *P*2_1_/*c*	Triclinic, *P* 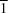
Temperature (K)	298	296
*a*, *b*, *c* (Å)	14.7179 (7), 7.5783 (4), 11.5451 (5)	9.0150 (4), 14.0601 (6), 16.2611 (8)
α, β, γ (°)	90, 102.999 (4), 90	105.146 (4), 95.967 (4), 98.057 (4)
*V* (Å^3^)	1254.70 (11)	1948.46 (16)
*Z*	4	4
Radiation type	Mo *K*α	Cu *K*α
μ (mm^−1^)	0.45	0.55
Crystal size (mm)	0.26 × 0.22 × 0.15	0.21 × 0.14 × 0.10

Data collection		
Diffractometer	Agilent Xcalibur Eos Gemini	Agilent Xcalibur Eos Gemini
Absorption correction	Multi-scan (*CrysAlis PRO*; Agilent, 2014 ▸)	Multi-scan (*CrysAlis PRO*; Agilent, 2014 ▸)
*T*_min_, *T*_max_	0.832, 0.935	0.737, 0.947
No. of measured, independent and observed [*I* > 2σ(*I*)] reflections	6097, 2785, 2053	12946, 7079, 5485
*R*_int_	0.028	0.035
(sin θ/λ)_max_ (Å^−1^)	0.651	0.601

Refinement		
*R*[*F* ^2^ > 2σ(*F* ^2^)], *wR*(*F* ^2^), *S*	0.040, 0.094, 1.04	0.050, 0.143, 1.04
No. of reflections	2785	7079
No. of parameters	156	575
No. of restraints	0	72
H-atom treatment	H-atom parameters constrained	H-atom parameters constrained
Δρ_max_, Δρ_min_ (e Å^−3^)	0.18, −0.19	0.22, −0.26

Computer programs: *CrysAlis PRO* (Agilent, 2014 ▸), *SHELXS97* (Sheldrick, 2008 ▸), *SHELXL2014* (Sheldrick, 2015 ▸) and *PLATON* (Spek, 2009 ▸).

## supplementary crystallographic information

### (I) (2E)-1-(5-Chlorothiophen-2-yl)-3-(2-methylphenyl)prop-2-en-1-one Crystal data

**Table:**

C_14_H_11_ClOS	*F*(000) = 544
*M**_r_* = 262.74	*D*_x_ = 1.391 Mg m^−^^3^
Monoclinic, *P*2_1_/*c*	Mo *K*α radiation, λ = 0.71073 Å
*a* = 14.7179 (7) Å	Cell parameters from 2913 reflections
*b* = 7.5783 (4) Å	θ = 3.0–29.4°
*c* = 11.5451 (5) Å	µ = 0.45 mm^−^^1^
β = 102.999 (4)°	*T* = 298 K
*V* = 1254.70 (11) Å^3^	Block, colourless
*Z* = 4	0.26 × 0.22 × 0.15 mm

### (I) (2E)-1-(5-Chlorothiophen-2-yl)-3-(2-methylphenyl)prop-2-en-1-one Data collection

**Table:**

Agilent Xcalibur Eos Gemini diffractometer	2053 reflections with *I* > 2σ(*I*)
Detector resolution: 16.0416 pixels mm^-1^	*R*_int_ = 0.028
φ and ω scans	θ_max_ = 27.6°, θ_min_ = 3.0°
Absorption correction: multi-scan (CrysAlis PRO; Agilent, 2014)	*h* = −17→18
*T*_min_ = 0.832, *T*_max_ = 0.935	*k* = −5→9
6097 measured reflections	*l* = −14→14
2785 independent reflections	

### (I) (2E)-1-(5-Chlorothiophen-2-yl)-3-(2-methylphenyl)prop-2-en-1-one Refinement

**Table:**

Refinement on *F*^2^	Hydrogen site location: inferred from neighbouring sites
Least-squares matrix: full	H-atom parameters constrained
*R*[*F*^2^ > 2σ(*F*^2^)] = 0.040	*w* = 1/[σ^2^(*F*_o_^2^) + (0.0304*P*)^2^ + 0.3588*P*] where *P* = (*F*_o_^2^ + 2*F*_c_^2^)/3
*wR*(*F*^2^) = 0.094	(Δ/σ)_max_ = 0.001
*S* = 1.04	Δρ_max_ = 0.18 e Å^−^^3^
2785 reflections	Δρ_min_ = −0.19 e Å^−^^3^
156 parameters	Extinction correction: SHELXL2014 (Sheldrick, 2015), Fc^*^=kFc[1+0.001xFc^2^λ^3^/sin(2θ)]^-1/4^
0 restraints	Extinction coefficient: 0.0028 (8)

### (I) (2E)-1-(5-Chlorothiophen-2-yl)-3-(2-methylphenyl)prop-2-en-1-one Special details

**Table:**

Geometry. All esds (except the esd in the dihedral angle between two l.s. planes) are estimated using the full covariance matrix. The cell esds are taken into account individually in the estimation of esds in distances, angles and torsion angles; correlations between esds in cell parameters are only used when they are defined by crystal symmetry. An approximate (isotropic) treatment of cell esds is used for estimating esds involving l.s. planes.

### (I) (2E)-1-(5-Chlorothiophen-2-yl)-3-(2-methylphenyl)prop-2-en-1-one Fractional atomic coordinates and isotropic or equivalent isotropic displacement parameters (Å^2^)

**Table:**

	*x*	*y*	*z*	*U*_iso_*/*U*_eq_	
C1	0.43382 (14)	0.2903 (3)	0.56191 (17)	0.0443 (5)	
O1	0.41785 (10)	0.3013 (2)	0.66164 (12)	0.0649 (5)	
C2	0.36322 (13)	0.3328 (3)	0.45350 (17)	0.0451 (5)	
H2	0.3771	0.3155	0.3796	0.054*	
C3	0.28068 (14)	0.3950 (3)	0.45918 (17)	0.0458 (5)	
H3	0.2706	0.4129	0.5350	0.055*	
S11	0.60913 (4)	0.18403 (8)	0.67339 (4)	0.04994 (18)	
C12	0.52502 (13)	0.2307 (3)	0.54715 (16)	0.0398 (5)	
C13	0.55665 (13)	0.1979 (3)	0.44730 (16)	0.0441 (5)	
H13	0.5207	0.2148	0.3709	0.053*	
C14	0.64871 (14)	0.1362 (3)	0.47103 (17)	0.0485 (5)	
H14	0.6808	0.1076	0.4128	0.058*	
C15	0.68504 (13)	0.1233 (3)	0.58937 (17)	0.0434 (5)	
Cl15	0.79457 (4)	0.05248 (8)	0.65700 (5)	0.0609 (2)	
C31	0.20305 (13)	0.4390 (3)	0.35977 (16)	0.0418 (5)	
C32	0.12392 (14)	0.5248 (3)	0.38106 (19)	0.0474 (5)	
C33	0.05096 (15)	0.5622 (3)	0.2844 (2)	0.0569 (6)	
H33	−0.0020	0.6185	0.2974	0.068*	
C34	0.05518 (16)	0.5185 (3)	0.1710 (2)	0.0623 (7)	
H34	0.0054	0.5449	0.1081	0.075*	
C35	0.13302 (15)	0.4352 (3)	0.14948 (19)	0.0588 (6)	
H35	0.1362	0.4061	0.0722	0.071*	
C36	0.20586 (14)	0.3957 (3)	0.24326 (17)	0.0499 (5)	
H36	0.2582	0.3388	0.2287	0.060*	
C37	0.11721 (16)	0.5819 (4)	0.5038 (2)	0.0667 (7)	
H37A	0.1232	0.4808	0.5550	0.100*	
H37B	0.0579	0.6372	0.4999	0.100*	
H37C	0.1662	0.6643	0.5347	0.100*	

### (I) (2E)-1-(5-Chlorothiophen-2-yl)-3-(2-methylphenyl)prop-2-en-1-one Atomic displacement parameters (Å^2^)

**Table:**

	*U*^11^	*U*^22^	*U*^33^	*U*^12^	*U*^13^	*U*^23^
C1	0.0463 (11)	0.0518 (13)	0.0340 (10)	−0.0035 (10)	0.0071 (8)	−0.0033 (9)
O1	0.0549 (9)	0.1060 (14)	0.0336 (8)	0.0117 (9)	0.0097 (6)	−0.0032 (8)
C2	0.0449 (11)	0.0563 (13)	0.0330 (10)	−0.0021 (10)	0.0063 (8)	−0.0011 (9)
C3	0.0497 (12)	0.0521 (13)	0.0365 (10)	0.0002 (10)	0.0113 (8)	−0.0001 (9)
S11	0.0493 (3)	0.0704 (4)	0.0272 (2)	0.0019 (3)	0.0024 (2)	−0.0003 (2)
C12	0.0404 (10)	0.0463 (12)	0.0304 (9)	−0.0043 (9)	0.0031 (7)	0.0003 (8)
C13	0.0439 (11)	0.0570 (13)	0.0293 (9)	−0.0010 (10)	0.0039 (8)	0.0033 (9)
C14	0.0465 (11)	0.0648 (14)	0.0350 (10)	0.0018 (11)	0.0107 (8)	0.0006 (10)
C15	0.0406 (10)	0.0469 (12)	0.0405 (11)	−0.0031 (10)	0.0042 (8)	0.0019 (9)
Cl15	0.0479 (3)	0.0704 (4)	0.0579 (4)	0.0077 (3)	−0.0016 (2)	0.0077 (3)
C31	0.0420 (11)	0.0417 (11)	0.0407 (11)	−0.0055 (9)	0.0074 (8)	0.0037 (9)
C32	0.0450 (11)	0.0456 (12)	0.0523 (12)	−0.0067 (10)	0.0124 (9)	0.0000 (10)
C33	0.0407 (11)	0.0606 (15)	0.0679 (15)	0.0019 (11)	0.0090 (10)	0.0045 (12)
C34	0.0505 (13)	0.0740 (17)	0.0557 (14)	−0.0022 (13)	−0.0021 (10)	0.0132 (13)
C35	0.0564 (13)	0.0760 (17)	0.0413 (12)	−0.0036 (13)	0.0048 (9)	0.0076 (11)
C36	0.0469 (11)	0.0598 (14)	0.0425 (11)	0.0013 (11)	0.0090 (9)	0.0039 (10)
C37	0.0559 (14)	0.0811 (19)	0.0661 (16)	0.0043 (13)	0.0199 (11)	−0.0140 (14)

### (I) (2E)-1-(5-Chlorothiophen-2-yl)-3-(2-methylphenyl)prop-2-en-1-one Geometric parameters (Å, º)

**Table:**

C1—O1	1.229 (2)	C31—C36	1.394 (3)
C1—C12	1.462 (3)	C31—C32	1.403 (3)
C1—C2	1.471 (3)	C32—C33	1.393 (3)
C2—C3	1.318 (3)	C32—C37	1.506 (3)
C2—H2	0.9300	C33—C34	1.365 (3)
C3—C31	1.464 (3)	C33—H33	0.9300
C3—H3	0.9300	C34—C35	1.379 (3)
S11—C15	1.700 (2)	C34—H34	0.9300
S11—C12	1.7234 (18)	C35—C36	1.375 (3)
C12—C13	1.360 (3)	C35—H35	0.9300
C13—C14	1.401 (3)	C36—H36	0.9300
C13—H13	0.9300	C37—H37A	0.9600
C14—C15	1.354 (3)	C37—H37B	0.9600
C14—H14	0.9300	C37—H37C	0.9600
C15—Cl15	1.7118 (19)		
			
O1—C1—C12	120.29 (17)	C36—C31—C3	121.03 (18)
O1—C1—C2	122.34 (18)	C32—C31—C3	120.03 (18)
C12—C1—C2	117.37 (17)	C33—C32—C31	118.3 (2)
C3—C2—C1	121.22 (18)	C33—C32—C37	119.6 (2)
C3—C2—H2	119.4	C31—C32—C37	122.06 (18)
C1—C2—H2	119.4	C34—C33—C32	121.7 (2)
C2—C3—C31	127.43 (18)	C34—C33—H33	119.1
C2—C3—H3	116.3	C32—C33—H33	119.1
C31—C3—H3	116.3	C33—C34—C35	120.2 (2)
C15—S11—C12	90.70 (9)	C33—C34—H34	119.9
C13—C12—C1	130.82 (17)	C35—C34—H34	119.9
C13—C12—S11	111.20 (14)	C36—C35—C34	119.4 (2)
C1—C12—S11	117.94 (14)	C36—C35—H35	120.3
C12—C13—C14	113.31 (17)	C34—C35—H35	120.3
C12—C13—H13	123.3	C35—C36—C31	121.4 (2)
C14—C13—H13	123.3	C35—C36—H36	119.3
C15—C14—C13	111.50 (18)	C31—C36—H36	119.3
C15—C14—H14	124.3	C32—C37—H37A	109.5
C13—C14—H14	124.3	C32—C37—H37B	109.5
C14—C15—S11	113.28 (15)	H37A—C37—H37B	109.5
C14—C15—Cl15	126.91 (17)	C32—C37—H37C	109.5
S11—C15—Cl15	119.81 (11)	H37A—C37—H37C	109.5
C36—C31—C32	118.95 (18)	H37B—C37—H37C	109.5
			
O1—C1—C2—C3	4.0 (3)	C12—S11—C15—Cl15	−179.75 (14)
C12—C1—C2—C3	−176.9 (2)	C2—C3—C31—C36	8.6 (3)
C1—C2—C3—C31	−178.39 (19)	C2—C3—C31—C32	−171.9 (2)
O1—C1—C12—C13	175.4 (2)	C36—C31—C32—C33	0.4 (3)
C2—C1—C12—C13	−3.7 (3)	C3—C31—C32—C33	−179.11 (19)
O1—C1—C12—S11	−2.0 (3)	C36—C31—C32—C37	−177.6 (2)
C2—C1—C12—S11	178.92 (15)	C3—C31—C32—C37	2.8 (3)
C15—S11—C12—C13	0.45 (17)	C31—C32—C33—C34	−0.4 (3)
C15—S11—C12—C1	178.33 (17)	C37—C32—C33—C34	177.7 (2)
C1—C12—C13—C14	−177.9 (2)	C32—C33—C34—C35	−0.1 (4)
S11—C12—C13—C14	−0.4 (2)	C33—C34—C35—C36	0.5 (4)
C12—C13—C14—C15	0.1 (3)	C34—C35—C36—C31	−0.4 (4)
C13—C14—C15—S11	0.3 (3)	C32—C31—C36—C35	0.0 (3)
C13—C14—C15—Cl15	179.56 (17)	C3—C31—C36—C35	179.5 (2)
C12—S11—C15—C14	−0.43 (18)		

### (I) (2E)-1-(5-Chlorothiophen-2-yl)-3-(2-methylphenyl)prop-2-en-1-one Hydrogen-bond geometry (Å, º)

**Table:**

*D*—H···*A*	*D*—H	H···*A*	*D*···*A*	*D*—H···*A*
C13—H13···O1^i^	0.93	2.55	3.467 (2)	169

Symmetry code: (i) *x*, −*y*+1/2, *z*−1/2.

### (II) (2E)-1-(Anthracen-9-yl)-3-[4-(propan-2-yl)phenyl]prop-2-en-1-one Crystal data

**Table:**

C_26_H_22_O	*Z* = 4
*M**_r_* = 350.44	*F*(000) = 744
Triclinic, *P*1	*D*_x_ = 1.195 Mg m^−^^3^
*a* = 9.0150 (4) Å	Cu *K*α radiation, λ = 1.54184 Å
*b* = 14.0601 (6) Å	Cell parameters from 7095 reflections
*c* = 16.2611 (8) Å	θ = 3.5–68.0°
α = 105.146 (4)°	µ = 0.55 mm^−^^1^
β = 95.967 (4)°	*T* = 296 K
γ = 98.057 (4)°	Block, colourless
*V* = 1948.46 (16) Å^3^	0.21 × 0.14 × 0.10 mm

### (II) (2E)-1-(Anthracen-9-yl)-3-[4-(propan-2-yl)phenyl]prop-2-en-1-one Data collection

**Table:**

Agilent Xcalibur Eos Gemini diffractometer	5485 reflections with *I* > 2σ(*I*)
Detector resolution: 16.0416 pixels mm^-1^	*R*_int_ = 0.035
φ and ω scans	θ_max_ = 68.0°, θ_min_ = 3.3°
Absorption correction: multi-scan (CrysAlis PRO; Agilent, 2014)	*h* = −10→10
*T*_min_ = 0.737, *T*_max_ = 0.947	*k* = −16→15
12946 measured reflections	*l* = −19→17
7079 independent reflections	

### (II) (2E)-1-(Anthracen-9-yl)-3-[4-(propan-2-yl)phenyl]prop-2-en-1-one Refinement

**Table:**

Refinement on *F*^2^	72 restraints
Least-squares matrix: full	Hydrogen site location: inferred from neighbouring sites
*R*[*F*^2^ > 2σ(*F*^2^)] = 0.050	H-atom parameters constrained
*wR*(*F*^2^) = 0.143	*w* = 1/[σ^2^(*F*_o_^2^) + (0.0689*P*)^2^ + 0.2921*P*] where *P* = (*F*_o_^2^ + 2*F*_c_^2^)/3
*S* = 1.04	(Δ/σ)_max_ < 0.001
7079 reflections	Δρ_max_ = 0.22 e Å^−^^3^
575 parameters	Δρ_min_ = −0.26 e Å^−^^3^

### (II) (2E)-1-(Anthracen-9-yl)-3-[4-(propan-2-yl)phenyl]prop-2-en-1-one Special details

**Table:**

Geometry. All esds (except the esd in the dihedral angle between two l.s. planes) are estimated using the full covariance matrix. The cell esds are taken into account individually in the estimation of esds in distances, angles and torsion angles; correlations between esds in cell parameters are only used when they are defined by crystal symmetry. An approximate (isotropic) treatment of cell esds is used for estimating esds involving l.s. planes.

### (II) (2E)-1-(Anthracen-9-yl)-3-[4-(propan-2-yl)phenyl]prop-2-en-1-one Fractional atomic coordinates and isotropic or equivalent isotropic displacement parameters (Å^2^)

**Table:**

	*x*	*y*	*z*	*U*_iso_*/*U*_eq_	Occ. (<1)
C11	0.71500 (19)	0.10252 (14)	0.34353 (11)	0.0392 (4)	
O11	0.80294 (15)	0.04914 (12)	0.31426 (9)	0.0570 (4)	
C12	0.55261 (18)	0.08287 (13)	0.30901 (11)	0.0390 (4)	
H12	0.4899	0.1246	0.3359	0.047*	
C13	0.49356 (18)	0.00672 (13)	0.24008 (10)	0.0366 (4)	
H13	0.5608	−0.0324	0.2149	0.044*	
C111	0.8247 (3)	0.08400 (15)	0.51371 (13)	0.0549 (5)	
H111	0.7926	0.0267	0.4678	0.066*	
C112	0.8752 (3)	0.07482 (18)	0.59246 (15)	0.0702 (7)	
H112	0.8780	0.0116	0.5997	0.084*	
C113	0.9235 (3)	0.16040 (19)	0.66349 (14)	0.0706 (7)	
H113	0.9579	0.1533	0.7171	0.085*	
C114	0.9198 (2)	0.25229 (17)	0.65374 (12)	0.0554 (5)	
H114	0.9512	0.3079	0.7012	0.066*	
C115	0.8165 (2)	0.47150 (15)	0.47098 (14)	0.0509 (5)	
H115	0.8472	0.5275	0.5182	0.061*	
C116	0.7706 (2)	0.48406 (17)	0.39327 (16)	0.0591 (5)	
H116	0.7697	0.5481	0.3876	0.071*	
C117	0.7240 (2)	0.39997 (17)	0.32061 (14)	0.0534 (5)	
H117	0.6926	0.4091	0.2673	0.064*	
C118	0.72458 (19)	0.30582 (15)	0.32776 (12)	0.0434 (4)	
H118	0.6952	0.2515	0.2790	0.052*	
C119	0.76971 (17)	0.19321 (13)	0.41974 (10)	0.0350 (3)	
C120	0.86656 (18)	0.36079 (13)	0.56169 (11)	0.0413 (4)	
H120	0.8978	0.4166	0.6091	0.050*	
C121	0.81986 (19)	0.17963 (13)	0.49995 (11)	0.0397 (4)	
C122	0.86911 (19)	0.26655 (14)	0.57270 (11)	0.0406 (4)	
C123	0.81887 (17)	0.37492 (13)	0.48214 (11)	0.0388 (4)	
C124	0.76959 (16)	0.28911 (13)	0.40878 (10)	0.0354 (4)	
C131	0.33555 (18)	−0.02239 (12)	0.19949 (10)	0.0334 (3)	
C132	0.22085 (19)	0.02522 (13)	0.23310 (11)	0.0388 (4)	
H132	0.2444	0.0772	0.2839	0.047*	
C133	0.07321 (19)	−0.00304 (14)	0.19272 (11)	0.0415 (4)	
H133	−0.0010	0.0301	0.2168	0.050*	
C134	0.03292 (18)	−0.08023 (12)	0.11669 (10)	0.0346 (3)	
C135	0.14636 (19)	−0.12848 (13)	0.08311 (10)	0.0379 (4)	
H135	0.1222	−0.1806	0.0325	0.045*	
C136	0.29520 (19)	−0.10051 (13)	0.12369 (10)	0.0384 (4)	
H136	0.3691	−0.1343	0.1000	0.046*	
C137	−0.12965 (19)	−0.11137 (14)	0.07197 (11)	0.0418 (4)	
H137	−0.1283	−0.1496	0.0124	0.050*	
C138	−0.2217 (2)	−0.17970 (16)	0.11443 (17)	0.0579 (5)	
H18A	−0.1753	−0.2374	0.1131	0.087*	
H18B	−0.3229	−0.2006	0.0838	0.087*	
H18C	−0.2247	−0.1441	0.1731	0.087*	
C139	−0.2078 (2)	−0.02305 (17)	0.06944 (15)	0.0592 (6)	
H19A	−0.2207	0.0115	0.1268	0.089*	
H19B	−0.3050	−0.0466	0.0342	0.089*	
H19C	−0.1469	0.0218	0.0457	0.089*	
C21	0.9228 (7)	0.7212 (7)	0.8184 (4)	0.0348 (4)	0.644 (3)
O21	1.039 (2)	0.767 (3)	0.8666 (11)	0.0455 (13)	0.644 (3)
C22	0.8944 (14)	0.7333 (14)	0.7316 (7)	0.0352 (12)	0.644 (3)
H22	0.9723	0.7693	0.7131	0.042*	0.644 (3)
C23	0.7644 (14)	0.6963 (12)	0.6766 (6)	0.0345 (14)	0.644 (3)
H23	0.6905	0.6548	0.6930	0.041*	0.644 (3)
C211	1.0065 (10)	0.5367 (9)	0.8411 (12)	0.0385 (13)	0.644 (3)
H211	1.0698	0.5772	0.8175	0.046*	0.644 (3)
C212	1.0495 (10)	0.4529 (8)	0.8551 (10)	0.0470 (11)	0.644 (3)
H212	1.1438	0.4383	0.8434	0.056*	0.644 (3)
C213	0.9526 (6)	0.3874 (6)	0.8874 (8)	0.0501 (13)	0.644 (3)
H213	0.9858	0.3322	0.8996	0.060*	0.644 (3)
C214	0.8124 (7)	0.4050 (6)	0.9006 (9)	0.0455 (12)	0.644 (3)
H214	0.7470	0.3587	0.9173	0.055*	0.644 (3)
C215	0.4326 (8)	0.6279 (5)	0.9197 (7)	0.0391 (10)	0.644 (3)
H215	0.3670	0.5831	0.9380	0.047*	0.644 (3)
C216	0.3911 (8)	0.7152 (4)	0.9141 (7)	0.0413 (12)	0.644 (3)
H216	0.2972	0.7292	0.9275	0.050*	0.644 (3)
C217	0.4909 (10)	0.7852 (5)	0.8880 (9)	0.0407 (10)	0.644 (3)
H217	0.4633	0.8461	0.8862	0.049*	0.644 (3)
C218	0.6263 (12)	0.7646 (6)	0.8655 (13)	0.0365 (10)	0.644 (3)
H218	0.6882	0.8104	0.8463	0.044*	0.644 (3)
C219	0.8136 (8)	0.6487 (6)	0.8467 (7)	0.0312 (9)	0.644 (3)
C220	0.6222 (7)	0.5163 (5)	0.9079 (7)	0.0375 (9)	0.644 (3)
H220	0.5581	0.4720	0.9275	0.045*	0.644 (3)
C221	0.8648 (10)	0.5636 (9)	0.8621 (13)	0.0327 (11)	0.644 (3)
C222	0.7624 (10)	0.4933 (8)	0.8892 (12)	0.0366 (8)	0.644 (3)
C223	0.5748 (10)	0.6032 (6)	0.8982 (10)	0.0339 (8)	0.644 (3)
C224	0.6750 (13)	0.6737 (9)	0.8708 (15)	0.0322 (6)	0.644 (3)
C231	0.7290 (9)	0.7158 (7)	0.5930 (4)	0.0336 (11)	0.644 (3)
C232	0.8287 (5)	0.7781 (5)	0.5618 (3)	0.0379 (7)	0.644 (3)
H232	0.9225	0.8087	0.5942	0.046*	0.644 (3)
C233	0.7891 (4)	0.7948 (3)	0.4830 (2)	0.0399 (7)	0.644 (3)
H233	0.8569	0.8372	0.4636	0.048*	0.644 (3)
C234	0.6504 (4)	0.7499 (3)	0.43184 (18)	0.0400 (7)	0.644 (3)
C235	0.5513 (4)	0.6873 (3)	0.4631 (2)	0.0434 (8)	0.644 (3)
H235	0.4581	0.6559	0.4303	0.052*	0.644 (3)
C236	0.5898 (4)	0.6713 (3)	0.5423 (2)	0.0393 (8)	0.644 (3)
H236	0.5212	0.6298	0.5622	0.047*	0.644 (3)
C237	0.6140 (3)	0.7666 (3)	0.34365 (19)	0.0523 (7)	0.644 (3)
H237	0.6886	0.8228	0.3403	0.063*	0.644 (3)
C238	0.630 (3)	0.6736 (15)	0.2717 (5)	0.0767 (12)	0.644 (3)
H28A	0.6142	0.6872	0.2169	0.115*	0.644 (3)
H28B	0.5550	0.6179	0.2722	0.115*	0.644 (3)
H28C	0.7289	0.6578	0.2813	0.115*	0.644 (3)
C239	0.4590 (6)	0.7932 (5)	0.3291 (4)	0.105 (2)	0.644 (3)
H29A	0.4493	0.8494	0.3754	0.158*	0.644 (3)
H29B	0.3832	0.7371	0.3270	0.158*	0.644 (3)
H29C	0.4460	0.8101	0.2756	0.158*	0.644 (3)
C31	0.9247 (13)	0.7242 (12)	0.8178 (7)	0.0348 (4)	0.356 (3)
O31	1.047 (4)	0.767 (5)	0.861 (2)	0.0455 (13)	0.356 (3)
C32	0.884 (3)	0.737 (3)	0.7321 (12)	0.0352 (12)	0.356 (3)
H32	0.9451	0.7860	0.7159	0.042*	0.356 (3)
C33	0.763 (3)	0.683 (2)	0.6756 (12)	0.0345 (14)	0.356 (3)
H33	0.7045	0.6333	0.6924	0.041*	0.356 (3)
C311	0.9927 (19)	0.5405 (17)	0.851 (2)	0.0385 (13)	0.356 (3)
H311	1.0682	0.5840	0.8382	0.046*	0.356 (3)
C312	1.0239 (18)	0.4537 (15)	0.8647 (19)	0.0470 (11)	0.356 (3)
H312	1.1206	0.4386	0.8608	0.056*	0.356 (3)
C313	0.9114 (16)	0.3855 (12)	0.8845 (17)	0.0501 (13)	0.356 (3)
H313	0.9311	0.3233	0.8876	0.060*	0.356 (3)
C314	0.7757 (16)	0.4111 (12)	0.8990 (17)	0.0455 (12)	0.356 (3)
H314	0.7050	0.3678	0.9157	0.055*	0.356 (3)
C315	0.4254 (15)	0.6532 (10)	0.9175 (15)	0.0391 (10)	0.356 (3)
H315	0.3529	0.6096	0.9326	0.047*	0.356 (3)
C316	0.3952 (16)	0.7435 (10)	0.9131 (14)	0.0413 (12)	0.356 (3)
H316	0.3042	0.7624	0.9273	0.050*	0.356 (3)
C317	0.5024 (19)	0.8090 (11)	0.8869 (18)	0.0407 (10)	0.356 (3)
H317	0.4805	0.8706	0.8833	0.049*	0.356 (3)
C318	0.637 (2)	0.7833 (14)	0.867 (2)	0.0365 (10)	0.356 (3)
H318	0.7070	0.8287	0.8523	0.044*	0.356 (3)
C319	0.8138 (15)	0.6609 (12)	0.8538 (13)	0.0312 (9)	0.356 (3)
C320	0.5989 (14)	0.5309 (10)	0.9031 (15)	0.0375 (9)	0.356 (3)
H320	0.5247	0.4858	0.9155	0.045*	0.356 (3)
C321	0.845 (2)	0.5661 (16)	0.857 (3)	0.0327 (11)	0.356 (3)
C322	0.7383 (19)	0.5032 (15)	0.889 (2)	0.0366 (8)	0.356 (3)
C323	0.5666 (19)	0.6235 (12)	0.899 (2)	0.0339 (8)	0.356 (3)
C324	0.671 (2)	0.6876 (16)	0.868 (3)	0.0322 (6)	0.356 (3)
C331	0.7119 (16)	0.6938 (15)	0.5904 (8)	0.0336 (11)	0.356 (3)
C332	0.7896 (11)	0.7611 (10)	0.5540 (6)	0.0379 (7)	0.356 (3)
H332	0.8793	0.8019	0.5842	0.046*	0.356 (3)
C333	0.7358 (7)	0.7684 (6)	0.4732 (4)	0.0399 (7)	0.356 (3)
H333	0.7904	0.8136	0.4500	0.048*	0.356 (3)
C334	0.6015 (7)	0.7093 (5)	0.4264 (3)	0.0400 (7)	0.356 (3)
C335	0.5251 (7)	0.6414 (5)	0.4627 (4)	0.0434 (8)	0.356 (3)
H335	0.4364	0.5998	0.4320	0.052*	0.356 (3)
C336	0.5774 (8)	0.6341 (6)	0.5432 (4)	0.0393 (8)	0.356 (3)
H336	0.5224	0.5889	0.5663	0.047*	0.356 (3)
C337	0.5407 (6)	0.7158 (4)	0.3371 (3)	0.0523 (7)	0.356 (3)
H337	0.4377	0.6773	0.3196	0.063*	0.356 (3)
C338	0.643 (5)	0.671 (3)	0.2714 (9)	0.0767 (12)	0.356 (3)
H38A	0.6069	0.6779	0.2159	0.115*	0.356 (3)
H38B	0.6394	0.6015	0.2677	0.115*	0.356 (3)
H33C	0.7450	0.7061	0.2899	0.115*	0.356 (3)
C339	0.5370 (11)	0.8232 (7)	0.3378 (7)	0.105 (2)	0.356 (3)
H39A	0.4816	0.8247	0.2846	0.158*	0.356 (3)
H39B	0.6387	0.8584	0.3443	0.158*	0.356 (3)
H39C	0.4885	0.8546	0.3849	0.158*	0.356 (3)

### (II) (2E)-1-(Anthracen-9-yl)-3-[4-(propan-2-yl)phenyl]prop-2-en-1-one Atomic displacement parameters (Å^2^)

**Table:**

	*U*^11^	*U*^22^	*U*^33^	*U*^12^	*U*^13^	*U*^23^
C11	0.0341 (8)	0.0458 (10)	0.0339 (8)	0.0065 (7)	0.0038 (6)	0.0054 (7)
O11	0.0388 (7)	0.0710 (10)	0.0470 (7)	0.0172 (6)	0.0010 (5)	−0.0098 (6)
C12	0.0333 (8)	0.0402 (10)	0.0385 (8)	0.0069 (7)	0.0029 (6)	0.0030 (7)
C13	0.0340 (8)	0.0394 (9)	0.0347 (8)	0.0080 (6)	0.0055 (6)	0.0060 (7)
C111	0.0733 (14)	0.0409 (11)	0.0443 (10)	0.0019 (9)	−0.0023 (9)	0.0096 (8)
C112	0.103 (2)	0.0526 (13)	0.0551 (12)	0.0093 (12)	−0.0021 (12)	0.0232 (11)
C113	0.0945 (18)	0.0703 (16)	0.0436 (11)	0.0048 (13)	−0.0097 (11)	0.0228 (11)
C114	0.0621 (12)	0.0557 (12)	0.0372 (9)	−0.0015 (9)	−0.0069 (8)	0.0054 (9)
C115	0.0426 (10)	0.0402 (10)	0.0669 (12)	0.0042 (8)	0.0052 (9)	0.0123 (9)
C116	0.0509 (11)	0.0526 (12)	0.0834 (15)	0.0114 (9)	0.0116 (10)	0.0340 (11)
C117	0.0444 (10)	0.0693 (14)	0.0582 (11)	0.0158 (9)	0.0121 (8)	0.0333 (11)
C118	0.0328 (8)	0.0566 (11)	0.0422 (9)	0.0081 (7)	0.0073 (7)	0.0155 (8)
C119	0.0260 (7)	0.0406 (9)	0.0340 (8)	0.0025 (6)	0.0033 (6)	0.0050 (7)
C120	0.0316 (8)	0.0402 (10)	0.0410 (9)	−0.0021 (7)	−0.0001 (6)	−0.0015 (7)
C121	0.0359 (8)	0.0411 (10)	0.0375 (8)	0.0006 (7)	0.0012 (6)	0.0078 (7)
C122	0.0345 (8)	0.0436 (10)	0.0371 (8)	−0.0012 (7)	0.0000 (6)	0.0061 (7)
C123	0.0262 (7)	0.0400 (9)	0.0477 (9)	0.0020 (6)	0.0050 (6)	0.0103 (7)
C124	0.0229 (7)	0.0436 (9)	0.0396 (8)	0.0042 (6)	0.0060 (6)	0.0117 (7)
C131	0.0341 (8)	0.0324 (8)	0.0321 (8)	0.0033 (6)	0.0033 (6)	0.0084 (6)
C132	0.0360 (8)	0.0368 (9)	0.0350 (8)	0.0009 (7)	0.0053 (6)	−0.0021 (7)
C133	0.0324 (8)	0.0441 (10)	0.0430 (9)	0.0065 (7)	0.0091 (7)	0.0024 (8)
C134	0.0345 (8)	0.0362 (9)	0.0323 (8)	−0.0001 (6)	0.0042 (6)	0.0120 (7)
C135	0.0407 (9)	0.0369 (9)	0.0304 (8)	0.0037 (7)	0.0020 (6)	0.0027 (7)
C136	0.0378 (9)	0.0386 (9)	0.0353 (8)	0.0097 (7)	0.0052 (6)	0.0029 (7)
C137	0.0336 (8)	0.0479 (10)	0.0382 (8)	0.0011 (7)	0.0013 (6)	0.0067 (7)
C138	0.0333 (9)	0.0473 (11)	0.0949 (16)	0.0004 (8)	0.0018 (9)	0.0294 (11)
C139	0.0498 (11)	0.0594 (13)	0.0692 (13)	0.0014 (9)	−0.0123 (10)	0.0320 (11)
C21	0.0289 (8)	0.0351 (10)	0.0393 (8)	0.0075 (6)	0.0055 (6)	0.0073 (7)
O21	0.0344 (19)	0.0502 (8)	0.046 (2)	−0.0007 (16)	−0.001 (2)	0.010 (2)
C22	0.0293 (18)	0.0388 (16)	0.0411 (9)	0.0091 (18)	0.0101 (9)	0.0138 (9)
C23	0.0330 (8)	0.036 (4)	0.0365 (8)	0.0082 (15)	0.0107 (6)	0.0093 (15)
C211	0.0384 (17)	0.0397 (13)	0.035 (4)	0.0100 (11)	0.0113 (12)	0.003 (2)
C212	0.044 (3)	0.0512 (12)	0.050 (4)	0.0248 (19)	0.018 (2)	0.0079 (15)
C213	0.055 (4)	0.0447 (12)	0.0589 (15)	0.025 (3)	0.014 (4)	0.0179 (11)
C214	0.053 (4)	0.0430 (15)	0.0487 (12)	0.017 (2)	0.018 (4)	0.0179 (11)
C215	0.0320 (11)	0.053 (4)	0.0346 (10)	0.0083 (18)	0.0064 (10)	0.015 (3)
C216	0.0313 (10)	0.055 (4)	0.0394 (10)	0.016 (2)	0.0055 (7)	0.011 (3)
C217	0.0403 (17)	0.044 (4)	0.0405 (10)	0.017 (3)	0.0026 (13)	0.012 (3)
C218	0.0358 (16)	0.039 (4)	0.0338 (11)	0.007 (2)	0.0021 (16)	0.009 (4)
C219	0.0310 (8)	0.035 (2)	0.0246 (17)	0.0045 (10)	0.0025 (8)	0.0036 (18)
C220	0.037 (2)	0.043 (2)	0.0362 (16)	0.0079 (12)	0.008 (2)	0.0168 (17)
C221	0.031 (2)	0.0375 (11)	0.027 (2)	0.0063 (14)	0.000 (3)	0.0055 (7)
C222	0.040 (3)	0.040 (2)	0.0331 (8)	0.0126 (13)	0.010 (3)	0.0109 (19)
C223	0.0322 (12)	0.039 (4)	0.0300 (9)	0.0087 (16)	0.0046 (10)	0.008 (3)
C224	0.0304 (9)	0.039 (3)	0.0270 (15)	0.0079 (14)	0.0006 (10)	0.010 (3)
C231	0.0298 (19)	0.037 (5)	0.0360 (9)	0.0092 (12)	0.0104 (9)	0.0099 (18)
C232	0.025 (3)	0.046 (3)	0.0436 (13)	0.0042 (16)	0.0064 (13)	0.0144 (15)
C233	0.032 (2)	0.044 (2)	0.0473 (13)	0.0029 (14)	0.0112 (14)	0.0195 (15)
C234	0.0310 (19)	0.047 (2)	0.0451 (12)	0.0090 (13)	0.0074 (13)	0.0159 (15)
C235	0.0305 (14)	0.056 (3)	0.0450 (11)	0.0028 (17)	0.0042 (10)	0.0187 (18)
C236	0.0304 (11)	0.048 (3)	0.0421 (10)	0.0039 (17)	0.0100 (8)	0.0164 (16)
C237	0.0462 (17)	0.062 (2)	0.0557 (15)	0.0058 (12)	0.0034 (13)	0.0323 (15)
C238	0.095 (4)	0.092 (2)	0.0423 (11)	0.0142 (16)	0.0016 (12)	0.0234 (12)
C239	0.083 (4)	0.170 (5)	0.116 (3)	0.066 (4)	0.028 (4)	0.105 (4)
C31	0.0289 (8)	0.0351 (10)	0.0393 (8)	0.0075 (6)	0.0055 (6)	0.0073 (7)
O31	0.0344 (19)	0.0502 (8)	0.046 (2)	−0.0007 (16)	−0.001 (2)	0.010 (2)
C32	0.0293 (18)	0.0388 (16)	0.0411 (9)	0.0091 (18)	0.0101 (9)	0.0138 (9)
C33	0.0330 (8)	0.036 (4)	0.0365 (8)	0.0082 (15)	0.0107 (6)	0.0093 (15)
C311	0.0384 (17)	0.0397 (13)	0.035 (4)	0.0100 (11)	0.0113 (12)	0.003 (2)
C312	0.044 (3)	0.0512 (12)	0.050 (4)	0.0248 (19)	0.018 (2)	0.0079 (15)
C313	0.055 (4)	0.0447 (12)	0.0589 (15)	0.025 (3)	0.014 (4)	0.0179 (11)
C314	0.053 (4)	0.0430 (15)	0.0487 (12)	0.017 (2)	0.018 (4)	0.0179 (11)
C315	0.0320 (11)	0.053 (4)	0.0346 (10)	0.0083 (18)	0.0064 (10)	0.015 (3)
C316	0.0313 (10)	0.055 (4)	0.0394 (10)	0.016 (2)	0.0055 (7)	0.011 (3)
C317	0.0403 (17)	0.044 (4)	0.0405 (10)	0.017 (3)	0.0026 (13)	0.012 (3)
C318	0.0358 (16)	0.039 (4)	0.0338 (11)	0.007 (2)	0.0021 (16)	0.009 (4)
C319	0.0310 (8)	0.035 (2)	0.0246 (17)	0.0045 (10)	0.0025 (8)	0.0036 (18)
C320	0.037 (2)	0.043 (2)	0.0362 (16)	0.0079 (12)	0.008 (2)	0.0168 (17)
C321	0.031 (2)	0.0375 (11)	0.027 (2)	0.0063 (14)	0.000 (3)	0.0055 (7)
C322	0.040 (3)	0.040 (2)	0.0331 (8)	0.0126 (13)	0.010 (3)	0.0109 (19)
C323	0.0322 (12)	0.039 (4)	0.0300 (9)	0.0087 (16)	0.0046 (10)	0.008 (3)
C324	0.0304 (9)	0.039 (3)	0.0270 (15)	0.0079 (14)	0.0006 (10)	0.010 (3)
C331	0.0298 (19)	0.037 (5)	0.0360 (9)	0.0092 (12)	0.0104 (9)	0.0099 (18)
C332	0.025 (3)	0.046 (3)	0.0436 (13)	0.0042 (16)	0.0064 (13)	0.0144 (15)
C333	0.032 (2)	0.044 (2)	0.0473 (13)	0.0029 (14)	0.0112 (14)	0.0195 (15)
C334	0.0310 (19)	0.047 (2)	0.0451 (12)	0.0090 (13)	0.0074 (13)	0.0159 (15)
C335	0.0305 (14)	0.056 (3)	0.0450 (11)	0.0028 (17)	0.0042 (10)	0.0187 (18)
C336	0.0304 (11)	0.048 (3)	0.0421 (10)	0.0039 (17)	0.0100 (8)	0.0164 (16)
C337	0.0462 (17)	0.062 (2)	0.0557 (15)	0.0058 (12)	0.0034 (13)	0.0323 (15)
C338	0.095 (4)	0.092 (2)	0.0423 (11)	0.0142 (16)	0.0016 (12)	0.0234 (12)
C339	0.083 (4)	0.170 (5)	0.116 (3)	0.066 (4)	0.028 (4)	0.105 (4)

### (II) (2E)-1-(Anthracen-9-yl)-3-[4-(propan-2-yl)phenyl]prop-2-en-1-one Geometric parameters (Å, º)

**Table:**

C11—O11	1.215 (2)	C219—C221	1.413 (3)
C11—C12	1.473 (2)	C220—C223	1.390 (3)
C11—C119	1.509 (2)	C220—C222	1.392 (3)
C12—C13	1.332 (2)	C220—H220	0.9300
C12—H12	0.9300	C221—C222	1.441 (3)
C13—C131	1.460 (2)	C223—C224	1.435 (3)
C13—H13	0.9300	C231—C232	1.393 (4)
C111—C112	1.358 (3)	C231—C236	1.394 (4)
C111—C121	1.425 (3)	C232—C233	1.383 (3)
C111—H111	0.9300	C232—H232	0.9300
C112—C113	1.412 (3)	C233—C234	1.394 (4)
C112—H112	0.9300	C233—H233	0.9300
C113—C114	1.347 (3)	C234—C235	1.392 (4)
C113—H113	0.9300	C234—C237	1.523 (3)
C114—C122	1.423 (3)	C235—C236	1.382 (3)
C114—H114	0.9300	C235—H235	0.9300
C115—C116	1.352 (3)	C236—H236	0.9300
C115—C123	1.419 (3)	C237—C239	1.507 (5)
C115—H115	0.9300	C237—C238	1.545 (11)
C116—C117	1.413 (3)	C237—H237	0.9800
C116—H116	0.9300	C238—H28A	0.9600
C117—C118	1.359 (3)	C238—H28B	0.9600
C117—H117	0.9300	C238—H28C	0.9600
C118—C124	1.426 (2)	C239—H29A	0.9600
C118—H118	0.9300	C239—H29B	0.9600
C119—C121	1.405 (2)	C239—H29C	0.9600
C119—C124	1.406 (2)	C31—O31	1.221 (4)
C120—C122	1.386 (3)	C31—C32	1.468 (4)
C120—C123	1.393 (3)	C31—C319	1.510 (4)
C120—H120	0.9300	C32—C33	1.345 (6)
C121—C122	1.440 (2)	C32—H32	0.9300
C123—C124	1.436 (2)	C33—C331	1.465 (4)
C131—C132	1.392 (2)	C33—H33	0.9300
C131—C136	1.395 (2)	C311—C312	1.356 (4)
C132—C133	1.376 (2)	C311—C321	1.433 (5)
C132—H132	0.9300	C311—H311	0.9300
C133—C134	1.390 (2)	C312—C313	1.417 (5)
C133—H133	0.9300	C312—H312	0.9300
C134—C135	1.386 (2)	C313—C314	1.349 (5)
C134—C137	1.517 (2)	C313—H313	0.9300
C135—C136	1.387 (2)	C314—C322	1.427 (4)
C135—H135	0.9300	C314—H314	0.9300
C136—H136	0.9300	C315—C316	1.353 (5)
C137—C139	1.518 (3)	C315—C323	1.430 (4)
C137—C138	1.526 (3)	C315—H315	0.9300
C137—H137	0.9800	C316—C317	1.416 (5)
C138—H18A	0.9600	C316—H316	0.9300
C138—H18B	0.9600	C317—C318	1.358 (4)
C138—H18C	0.9600	C317—H317	0.9300
C139—H19A	0.9600	C318—C324	1.429 (5)
C139—H19B	0.9600	C318—H318	0.9300
C139—H19C	0.9600	C319—C324	1.412 (4)
C21—O21	1.222 (3)	C319—C321	1.414 (5)
C21—C22	1.466 (3)	C320—C323	1.390 (4)
C21—C219	1.509 (3)	C320—C322	1.390 (4)
C22—C23	1.346 (5)	C320—H320	0.9300
C22—H22	0.9300	C321—C322	1.443 (4)
C23—C231	1.467 (3)	C323—C324	1.436 (4)
C23—H23	0.9300	C331—C332	1.390 (5)
C211—C212	1.356 (3)	C331—C336	1.396 (5)
C211—C221	1.432 (4)	C332—C333	1.387 (4)
C211—H211	0.9300	C332—H332	0.9300
C212—C213	1.417 (4)	C333—C334	1.390 (5)
C212—H212	0.9300	C333—H333	0.9300
C213—C214	1.350 (4)	C334—C335	1.389 (5)
C213—H213	0.9300	C334—C337	1.528 (4)
C214—C222	1.428 (3)	C335—C336	1.380 (5)
C214—H214	0.9300	C335—H335	0.9300
C215—C216	1.354 (4)	C336—H336	0.9300
C215—C223	1.430 (3)	C337—C339	1.511 (6)
C215—H215	0.9300	C337—C338	1.549 (13)
C216—C217	1.417 (4)	C337—H337	0.9800
C216—H216	0.9300	C338—H38A	0.9600
C217—C218	1.358 (3)	C338—H38B	0.9600
C217—H217	0.9300	C338—H33C	0.9600
C218—C224	1.428 (3)	C339—H39A	0.9600
C218—H218	0.9300	C339—H39B	0.9600
C219—C224	1.411 (3)	C339—H39C	0.9600
			
O11—C11—C12	123.20 (16)	C214—C222—C221	118.5 (2)
O11—C11—C119	120.18 (15)	C220—C223—C215	122.1 (3)
C12—C11—C119	116.61 (14)	C220—C223—C224	119.4 (3)
C13—C12—C11	121.47 (16)	C215—C223—C224	118.5 (2)
C13—C12—H12	119.3	C219—C224—C218	122.9 (3)
C11—C12—H12	119.3	C219—C224—C223	119.0 (2)
C12—C13—C131	127.53 (16)	C218—C224—C223	118.1 (2)
C12—C13—H13	116.2	C232—C231—C236	117.9 (2)
C131—C13—H13	116.2	C232—C231—C23	122.7 (3)
C112—C111—C121	121.36 (19)	C236—C231—C23	119.4 (3)
C112—C111—H111	119.3	C233—C232—C231	120.4 (3)
C121—C111—H111	119.3	C233—C232—H232	119.8
C111—C112—C113	120.6 (2)	C231—C232—H232	119.8
C111—C112—H112	119.7	C232—C233—C234	121.8 (3)
C113—C112—H112	119.7	C232—C233—H233	119.1
C114—C113—C112	120.14 (19)	C234—C233—H233	119.1
C114—C113—H113	119.9	C235—C234—C233	117.6 (2)
C112—C113—H113	119.9	C235—C234—C237	122.2 (3)
C113—C114—C122	121.74 (18)	C233—C234—C237	120.2 (3)
C113—C114—H114	119.1	C236—C235—C234	120.8 (3)
C122—C114—H114	119.1	C236—C235—H235	119.6
C116—C115—C123	121.54 (19)	C234—C235—H235	119.6
C116—C115—H115	119.2	C235—C236—C231	121.5 (3)
C123—C115—H115	119.2	C235—C236—H236	119.3
C115—C116—C117	120.09 (19)	C231—C236—H236	119.3
C115—C116—H116	120.0	C239—C237—C234	112.7 (3)
C117—C116—H116	120.0	C239—C237—C238	110.2 (9)
C118—C117—C116	120.72 (18)	C234—C237—C238	110.5 (4)
C118—C117—H117	119.6	C239—C237—H237	107.7
C116—C117—H117	119.6	C234—C237—H237	107.7
C117—C118—C124	121.02 (18)	C238—C237—H237	107.7
C117—C118—H118	119.5	C237—C238—H28A	109.5
C124—C118—H118	119.5	C237—C238—H28B	109.5
C121—C119—C124	121.64 (15)	H28A—C238—H28B	109.5
C121—C119—C11	119.09 (15)	C237—C238—H28C	109.5
C124—C119—C11	119.28 (14)	H28A—C238—H28C	109.5
C122—C120—C123	122.30 (15)	H28B—C238—H28C	109.5
C122—C120—H120	118.9	C237—C239—H29A	109.5
C123—C120—H120	118.9	C237—C239—H29B	109.5
C119—C121—C111	123.58 (16)	H29A—C239—H29B	109.5
C119—C121—C122	118.70 (16)	C237—C239—H29C	109.5
C111—C121—C122	117.71 (16)	H29A—C239—H29C	109.5
C120—C122—C114	122.24 (16)	H29B—C239—H29C	109.5
C120—C122—C121	119.33 (16)	O31—C31—C32	120.0 (5)
C114—C122—C121	118.43 (17)	O31—C31—C319	120.1 (5)
C120—C123—C115	122.21 (17)	C32—C31—C319	119.8 (5)
C120—C123—C124	119.17 (16)	C33—C32—C31	124.1 (7)
C115—C123—C124	118.62 (16)	C33—C32—H32	118.0
C119—C124—C118	123.16 (16)	C31—C32—H32	118.0
C119—C124—C123	118.85 (15)	C32—C33—C331	127.1 (7)
C118—C124—C123	117.99 (16)	C32—C33—H33	116.4
C132—C131—C136	117.53 (15)	C331—C33—H33	116.4
C132—C131—C13	122.65 (15)	C312—C311—C321	120.9 (5)
C136—C131—C13	119.81 (15)	C312—C311—H311	119.5
C133—C132—C131	121.31 (15)	C321—C311—H311	119.5
C133—C132—H132	119.3	C311—C312—C313	121.0 (5)
C131—C132—H132	119.3	C311—C312—H312	119.5
C132—C133—C134	121.27 (16)	C313—C312—H312	119.5
C132—C133—H133	119.4	C314—C313—C312	119.9 (5)
C134—C133—H133	119.4	C314—C313—H313	120.0
C135—C134—C133	117.76 (15)	C312—C313—H313	120.0
C135—C134—C137	120.87 (15)	C313—C314—C322	121.4 (5)
C133—C134—C137	121.37 (15)	C313—C314—H314	119.3
C134—C135—C136	121.19 (15)	C322—C314—H314	119.3
C134—C135—H135	119.4	C316—C315—C323	121.4 (5)
C136—C135—H135	119.4	C316—C315—H315	119.3
C135—C136—C131	120.92 (16)	C323—C315—H315	119.3
C135—C136—H136	119.5	C315—C316—C317	119.8 (5)
C131—C136—H136	119.5	C315—C316—H316	120.1
C134—C137—C139	113.05 (15)	C317—C316—H316	120.1
C134—C137—C138	110.88 (15)	C318—C317—C316	121.1 (5)
C139—C137—C138	109.53 (16)	C318—C317—H317	119.5
C134—C137—H137	107.7	C316—C317—H317	119.5
C139—C137—H137	107.7	C317—C318—C324	121.0 (5)
C138—C137—H137	107.7	C317—C318—H318	119.5
C137—C138—H18A	109.5	C324—C318—H318	119.5
C137—C138—H18B	109.5	C324—C319—C321	121.0 (5)
H18A—C138—H18B	109.5	C324—C319—C31	120.4 (5)
C137—C138—H18C	109.5	C321—C319—C31	117.7 (5)
H18A—C138—H18C	109.5	C323—C320—C322	122.3 (5)
H18B—C138—H18C	109.5	C323—C320—H320	118.9
C137—C139—H19A	109.5	C322—C320—H320	118.9
C137—C139—H19B	109.5	C319—C321—C311	122.2 (8)
H19A—C139—H19B	109.5	C319—C321—C322	118.6 (5)
C137—C139—H19C	109.5	C311—C321—C322	117.3 (6)
H19A—C139—H19C	109.5	C320—C322—C314	122.4 (5)
H19B—C139—H19C	109.5	C320—C322—C321	118.9 (4)
O21—C21—C22	119.9 (3)	C314—C322—C321	118.5 (4)
O21—C21—C219	119.8 (3)	C320—C323—C315	122.0 (5)
C22—C21—C219	120.2 (3)	C320—C323—C324	119.2 (4)
C23—C22—C21	125.1 (5)	C315—C323—C324	118.6 (4)
C23—C22—H22	117.4	C319—C324—C318	122.6 (6)
C21—C22—H22	117.4	C319—C324—C323	118.8 (5)
C22—C23—C231	125.7 (4)	C318—C324—C323	117.7 (5)
C22—C23—H23	117.1	C332—C331—C336	117.8 (4)
C231—C23—H23	117.1	C332—C331—C33	123.7 (5)
C212—C211—C221	120.8 (3)	C336—C331—C33	118.5 (5)
C212—C211—H211	119.6	C333—C332—C331	121.2 (5)
C221—C211—H211	119.6	C333—C332—H332	119.4
C211—C212—C213	120.9 (3)	C331—C332—H332	119.4
C211—C212—H212	119.5	C332—C333—C334	121.1 (5)
C213—C212—H212	119.5	C332—C333—H333	119.5
C214—C213—C212	120.2 (3)	C334—C333—H333	119.5
C214—C213—H213	119.9	C335—C334—C333	117.5 (4)
C212—C213—H213	119.9	C335—C334—C337	120.6 (5)
C213—C214—C222	121.3 (3)	C333—C334—C337	121.9 (5)
C213—C214—H214	119.4	C336—C335—C334	121.8 (4)
C222—C214—H214	119.4	C336—C335—H335	119.1
C216—C215—C223	121.5 (3)	C334—C335—H335	119.1
C216—C215—H215	119.3	C335—C336—C331	120.7 (5)
C223—C215—H215	119.3	C335—C336—H336	119.7
C215—C216—C217	120.0 (3)	C331—C336—H336	119.7
C215—C216—H216	120.0	C339—C337—C334	110.9 (5)
C217—C216—H216	120.0	C339—C337—C338	109.2 (12)
C218—C217—C216	120.8 (3)	C334—C337—C338	109.6 (6)
C218—C217—H217	119.6	C339—C337—H337	109.0
C216—C217—H217	119.6	C334—C337—H337	109.0
C217—C218—C224	121.1 (3)	C338—C337—H337	109.0
C217—C218—H218	119.4	C337—C338—H38A	109.5
C224—C218—H218	119.4	C337—C338—H38B	109.5
C224—C219—C221	120.7 (3)	H38A—C338—H38B	109.5
C224—C219—C21	120.6 (3)	C337—C338—H33C	109.5
C221—C219—C21	117.9 (3)	H38A—C338—H33C	109.5
C223—C220—C222	122.2 (3)	H38B—C338—H33C	109.5
C223—C220—H220	118.9	C337—C339—H39A	109.5
C222—C220—H220	118.9	C337—C339—H39B	109.5
C219—C221—C211	122.8 (3)	H39A—C339—H39B	109.5
C219—C221—C222	118.8 (3)	C337—C339—H39C	109.5
C211—C221—C222	117.8 (3)	H39A—C339—H39C	109.5
C220—C222—C214	122.3 (3)	H39B—C339—H39C	109.5
C220—C222—C221	119.1 (3)		
			
O11—C11—C12—C13	−3.2 (3)	C216—C215—C223—C220	176.6 (13)
C119—C11—C12—C13	177.72 (16)	C216—C215—C223—C224	0 (2)
C11—C12—C13—C131	179.25 (17)	C221—C219—C224—C218	172.8 (19)
C121—C111—C112—C113	0.5 (4)	C21—C219—C224—C218	4 (3)
C111—C112—C113—C114	0.0 (4)	C221—C219—C224—C223	−10 (3)
C112—C113—C114—C122	−0.5 (4)	C21—C219—C224—C223	−179.4 (14)
C123—C115—C116—C117	−0.2 (3)	C217—C218—C224—C219	179.0 (18)
C115—C116—C117—C118	0.0 (3)	C217—C218—C224—C223	2 (3)
C116—C117—C118—C124	1.1 (3)	C220—C223—C224—C219	6 (3)
O11—C11—C119—C121	−66.5 (2)	C215—C223—C224—C219	−178.0 (16)
C12—C11—C119—C121	112.60 (18)	C220—C223—C224—C218	−177.0 (18)
O11—C11—C119—C124	113.3 (2)	C215—C223—C224—C218	−1 (3)
C12—C11—C119—C124	−67.5 (2)	C22—C23—C231—C232	−1 (3)
C124—C119—C121—C111	−178.78 (18)	C22—C23—C231—C236	179.3 (19)
C11—C119—C121—C111	1.1 (3)	C236—C231—C232—C233	0.2 (15)
C124—C119—C121—C122	1.3 (2)	C23—C231—C232—C233	−179.3 (13)
C11—C119—C121—C122	−178.81 (15)	C231—C232—C233—C234	−0.6 (12)
C112—C111—C121—C119	179.5 (2)	C232—C233—C234—C235	0.3 (7)
C112—C111—C121—C122	−0.6 (3)	C232—C233—C234—C237	−177.3 (5)
C123—C120—C122—C114	179.13 (18)	C233—C234—C235—C236	0.4 (6)
C123—C120—C122—C121	−0.5 (3)	C237—C234—C235—C236	178.0 (3)
C113—C114—C122—C120	−179.2 (2)	C234—C235—C236—C231	−0.9 (8)
C113—C114—C122—C121	0.4 (3)	C232—C231—C236—C235	0.6 (14)
C119—C121—C122—C120	−0.3 (2)	C23—C231—C236—C235	−180.0 (12)
C111—C121—C122—C120	179.76 (18)	C235—C234—C237—C239	50.0 (5)
C119—C121—C122—C114	−179.99 (17)	C233—C234—C237—C239	−132.5 (5)
C111—C121—C122—C114	0.1 (3)	C235—C234—C237—C238	−73.9 (11)
C122—C120—C123—C115	−179.93 (17)	C233—C234—C237—C238	103.7 (12)
C122—C120—C123—C124	0.4 (3)	O31—C31—C32—C33	170 (6)
C116—C115—C123—C120	179.68 (18)	C319—C31—C32—C33	−12 (5)
C116—C115—C123—C124	−0.6 (3)	C31—C32—C33—C331	178 (3)
C121—C119—C124—C118	177.72 (15)	C321—C311—C312—C313	0 (5)
C11—C119—C124—C118	−2.1 (2)	C311—C312—C313—C314	−7 (5)
C121—C119—C124—C123	−1.4 (2)	C312—C313—C314—C322	4 (5)
C11—C119—C124—C123	178.69 (14)	C323—C315—C316—C317	2 (4)
C117—C118—C124—C119	178.90 (16)	C315—C316—C317—C318	−1 (4)
C117—C118—C124—C123	−1.9 (2)	C316—C317—C318—C324	3 (5)
C120—C123—C124—C119	0.6 (2)	O31—C31—C319—C324	116 (5)
C115—C123—C124—C119	−179.11 (15)	C32—C31—C319—C324	−61 (4)
C120—C123—C124—C118	−178.63 (15)	O31—C31—C319—C321	−75 (5)
C115—C123—C124—C118	1.7 (2)	C32—C31—C319—C321	107 (3)
C12—C13—C131—C132	−4.1 (3)	C324—C319—C321—C311	−174 (3)
C12—C13—C131—C136	175.54 (17)	C31—C319—C321—C311	18 (4)
C136—C131—C132—C133	−0.5 (3)	C324—C319—C321—C322	−10 (5)
C13—C131—C132—C133	179.11 (17)	C31—C319—C321—C322	−178 (3)
C131—C132—C133—C134	−0.1 (3)	C312—C311—C321—C319	172 (3)
C132—C133—C134—C135	0.5 (3)	C312—C311—C321—C322	8 (5)
C132—C133—C134—C137	−179.87 (17)	C323—C320—C322—C314	175 (3)
C133—C134—C135—C136	−0.3 (3)	C323—C320—C322—C321	−9 (5)
C137—C134—C135—C136	−179.92 (16)	C313—C314—C322—C320	180 (3)
C134—C135—C136—C131	−0.3 (3)	C313—C314—C322—C321	4 (5)
C132—C131—C136—C135	0.8 (3)	C319—C321—C322—C320	9 (5)
C13—C131—C136—C135	−178.92 (16)	C311—C321—C322—C320	174 (3)
C135—C134—C137—C139	−137.63 (18)	C319—C321—C322—C314	−175 (3)
C133—C134—C137—C139	42.8 (2)	C311—C321—C322—C314	−10 (5)
C135—C134—C137—C138	98.9 (2)	C322—C320—C323—C315	−177 (3)
C133—C134—C137—C138	−80.7 (2)	C322—C320—C323—C324	9 (5)
O21—C21—C22—C23	−172 (3)	C316—C315—C323—C320	−180 (3)
C219—C21—C22—C23	11 (3)	C316—C315—C323—C324	−6 (5)
C21—C22—C23—C231	173.8 (18)	C321—C319—C324—C318	179 (4)
C221—C211—C212—C213	3 (3)	C31—C319—C324—C318	−13 (5)
C211—C212—C213—C214	3 (2)	C321—C319—C324—C323	10 (5)
C212—C213—C214—C222	−5 (2)	C31—C319—C324—C323	178 (3)
C223—C215—C216—C217	−1 (2)	C317—C318—C324—C319	−175 (3)
C215—C216—C217—C218	2 (2)	C317—C318—C324—C323	−6 (6)
C216—C217—C218—C224	−3 (3)	C320—C323—C324—C319	−9 (5)
O21—C21—C219—C224	106 (3)	C315—C323—C324—C319	177 (3)
C22—C21—C219—C224	−77.3 (19)	C320—C323—C324—C318	−178 (3)
O21—C21—C219—C221	−64 (3)	C315—C323—C324—C318	7 (5)
C22—C21—C219—C221	113.1 (16)	C32—C33—C331—C332	3 (6)
C224—C219—C221—C211	−178.3 (18)	C32—C33—C331—C336	−176 (4)
C21—C219—C221—C211	−9 (2)	C336—C331—C332—C333	0 (3)
C224—C219—C221—C222	10 (2)	C33—C331—C332—C333	−180 (3)
C21—C219—C221—C222	−180.0 (13)	C331—C332—C333—C334	1 (2)
C212—C211—C221—C219	−178.4 (17)	C332—C333—C334—C335	−1.3 (14)
C212—C211—C221—C222	−7 (3)	C332—C333—C334—C337	−179.7 (10)
C223—C220—C222—C214	178.9 (15)	C333—C334—C335—C336	1.7 (12)
C223—C220—C222—C221	3 (3)	C337—C334—C335—C336	−179.9 (7)
C213—C214—C222—C220	−175.6 (15)	C334—C335—C336—C331	−1.4 (18)
C213—C214—C222—C221	1 (3)	C332—C331—C336—C335	1 (3)
C219—C221—C222—C220	−7 (3)	C33—C331—C336—C335	−180 (2)
C211—C221—C222—C220	−178.3 (17)	C335—C334—C337—C339	131.4 (7)
C219—C221—C222—C214	177.0 (16)	C333—C334—C337—C339	−50.3 (9)
C211—C221—C222—C214	5 (3)	C335—C334—C337—C338	−108 (2)
C222—C220—C223—C215	−178.2 (15)	C333—C334—C337—C338	70 (2)
C222—C220—C223—C224	−2 (3)		

### (II) (2E)-1-(Anthracen-9-yl)-3-[4-(propan-2-yl)phenyl]prop-2-en-1-one Hydrogen-bond geometry (Å, º)

*Cg*1 and *Cg*2 are the centroids of rings (C111–C114/C122/C111) and (C114–C118/C124/C113), respectively.

**Table:**

*D*—H···*A*	*D*—H	H···*A*	*D*···*A*	*D*—H···*A*
C133—H133···O11^i^	0.93	2.49	3.336 (2)	151
C216—H216···O21^i^	0.93	2.61	3.41 (2)	144
C216—H216···O31^i^	0.93	2.58	3.36 (4)	142
C316—H316···O21^i^	0.93	2.51	3.30 (3)	144
C316—H316···O31^i^	0.93	2.47	3.25 (4)	142
C233—H233···*Cg*1^ii^	0.93	2.64	3.355 (4)	134
C236—H236···*Cg*2^iii^	0.93	2.75	3.519 (4)	140
C336—H336···*Cg*2^iii^	0.93	2.80	3.354 (7)	119

Symmetry codes: (i) *x*−1, *y*, *z*; (ii) −*x*+2, −*y*+1, −*z*+1; (iii) −*x*+1, −*y*+1, −*z*+1.
